# Effect of exercise in pregnant rats with mild diabetes on the immunological system and biochemical profiles

**DOI:** 10.1186/1758-5996-7-S1-A73

**Published:** 2015-11-11

**Authors:** Thaigra de Sousa Soares, Rafaianne Queiroz de Moraes Souza, Thamires Ballarini Gratão, Verônyca Gonçalves Paula, Larissa Lopes da Cruz, Kleber Eduardo de Campos, Débora Cristina Damasceno, Gustavo Tadeu Volpato

**Affiliations:** 1 Universidade Federal de Mato Grosso, Barra do Garças, Brazil

## Background

The practice of exercise aiming diabetes control is common, also during pregnancy. However, the potential benefits and risks of exercise during pregnancy, complicated or not by diabetes, is unknown.

## Objective

To evaluate immunological and biochemical biomarkers of the moderate intensity physical exercise after embryo implantation in pregnant rats with mild diabetes.

## Materials and methods

The experimental severe diabetes was induced in newborn female Wistar rats in the first day of birth by intravenous injection of Streptozotocin in a dose of 100 mg/Kg. In adult life (110 days) the rats were submitted to oral glucose tolerance test (OGTT) to confirm the moderate diabetes. After its confirmation, rats were mated and randomly assigned to 4 experimental groups (minimum n=13 animals/group): Control: non-diabetic pregnant rats without exercise (sedentary); Exercise: non-diabetic pregnant rats exposed to exercise; Diabetic: diabetic pregnant rats without exercise; Diabetic Exercise: diabetic pregnant rats exposed to exercise. The moderate intensity exercise program was swimming, from the 7th to 20th days of pregnancy. On days 0 and 17 of pregnancy, it was performed OGTT. At day 21 of pregnancy, the rats were anesthetized and the blood collected to evaluate immunological and biochemical parameters. Maternal organs (heart, liver, spleen and kidneys) were removed and immediately weighed to obtain its relative weight. Analysis of variance followed by Tukey's test were used, and the differences were considered statistically significant when p< 0.05.

## Results

The exercise did not alter any parameters in non-diabetic exercised rats compared to control rats. The mild diabetes animals presented increase in glicemia, triglycerides, very-low-density lipoprotein (VLDL), and relative liver weight in relation to non-diabetic groups. The exercise in diabetic rats only increased the relative kidney weights.

## Conclusion

Exercise during pregnancy did not alter biochemical and immunological profiles in mild diabetes, although this practice did not affect de non-diabetic rats.

